# The Impact of Aeroform Tissue Expanders on the Outcomes of Implant-Based Breast Reconstruction; A Systematic Review and Meta-Analysis

**DOI:** 10.1007/s00266-022-02901-y

**Published:** 2022-05-13

**Authors:** Abdelrahman Awadeen, Mohamed Fareed, Ali Mohamed Elameen

**Affiliations:** 1 Department of Plastic and Reconstructive Surgery, Faculty of Medicine (Boys), Al-Azhar University, Nasr City, Al Mokhaym Al Daem, Gameat Al Azhar, Cairo, Egypt; 2 Department of Plastic and Reconstructive Surgery, El-Sahel Teaching Hospital, Cairo, Egypt

**Keywords:** AeroForm, Carbon dioxide, Breast reconstruction

## Abstract

**Background:**

Breast reconstruction can improve the quality of patients' lives by restoring the breasts' natural appearance. Saline-based tissue expanders are associated with significant drawbacks. The current systematic review and meta-analysis aimed to reveal the usability, safety, and economic burden of AeroForm-based tissue expanders for breast reconstruction.

**Methods:**

An extensive systematic literature review was implemented from inception to 9 December 2021. All clinical studies that included women with breast cancer subjected to AeroForm-based tissue expansion for breast reconstruction were included in the study.

**Results:**

This systematic review included eleven articles consisting of 748 patients. There were 1220 reconstructed breasts in which 530 (43.44%) breasts were reconstructed using AeroForm devices. AeroForm-based tissue expanders were associated with shorter duration to complete breast expansion (MD-35.22; 95% -46.65, -23.78;P<0.001) and complete reconstruction (MD-30.511; 95% -54.659, -6.636;P=0.013). The overall satisfaction rate of the aesthetic results of the AeroForm expanders was 81.4% (95%CI; 60.3% to 92.6%,P=0.006) and 64.6% (95%CI; 53.8% to 74%,P=0.008) for patients and surgeons. Patients subjected to saline-based breast reconstruction were 1.17 times at high risk to develop breast-related adverse events (RR1.17; 95% 0.86, 1.58; P=0.31). This includes a high risk of mastectomy flap necrosis (RR1.91; 95% 1.03, 3.55;P=0.04) and post-operative wound infection (RR 1.63; 95% 0.91, 2.91;P=0.1).

**Conclusion:**

AeroForm-based tissue expanders represent a new era of breast reconstruction. These devices provided an earlier transition to exchange for the permanent implant with a convenient and comfortable expansion process. This was associated with a high satisfaction rate for patients and surgeons.

**Level of Evidence III:**

This journal requires that authors assign a level of evidence to each article. For a full description of these Evidence-Based Medicine ratings, please refer to the Table of Contents or the online Instructions to Authors www.springer.com/00266.

**Supplementary Information:**

The online version contains supplementary material available at 10.1007/s00266-022-02901-y.

## Introduction

Breast cancer is the most encountered malignancy in women worldwide. There were approximately 2.3 million women newly diagnosed with breast cancer in 2020, contributing to more than 685,000 deaths globally [1, 2]. The increasing number of women with breast cancer is associated with an upsurge in patients seeking breast surgeries. Mastectomy could be a life-saving procedure for patients with breast cancer. However, it represents a traumatic experience associated with devastating consequences on the patients’ appearance, social life, and sexuality [3]. Breast reconstruction can improve the quality of patients’ life by restoring the natural appearance of the breasts. It is associated with better psychological interactions, sexual well-being, and self-confidence [4].

Breast reconstruction is a pillar of breast cancer treatment. It is one of the most performed reconstructive surgeries in the USA. In 2019, more than 100,000 women underwent breast reconstruction, representing 40% of women subjected to mastectomy [5, 6]. Breast reconstruction can be assorted based on the composition of the reconstructed breast into either autologous or implant-based reconstruction. The majority of reconstruction procedures are implant-based, in which more than 80% of patients are subjected. The implant may be silicon, saline, or double lumen based on the physical design [7]. Saline-based tissue expanders typically expands the skin through serial percutaneous injections into the tissue expander port. This is performed in the clinic weekly or biweekly throughout the post-operative period. This model of breast expansion is associated with significant drawbacks. This includes patient discomfort, interruption of daily life, prolonged expansion time, and the risks associated with repeated injections [8–10]. Furthermore, saline-based devices are associated with the asymmetrical appearance of the breasts and lack of patient autonomy. These shortcomings discouraged some women from undergoing implant-based breast reconstruction [11]. This highlighted the need for recent innovation for a more safe and effective expansion process for breast reconstruction.

The AeroForm tissue expansion system is a temporary implanted device formed of stainless steel containing a compressed carbon dioxide reservoir. It is a needle-free device that allows gradual expansion through a remote controller. This prevents the need for repeated subcutaneous injections associated with saline expanders. The controller device wirelessly provides the release of 10 ccs of carbon dioxide per dose. The expander is designed to release the gas from the internal reservoir up to the programmed volume of the expander. This mechanism allowed for a maximum of three expansions each day. The surgeons can execute additional volume expansion beyond the labelled limits on needs [12–14].

AeroForm-based breast reconstruction provides a unique experience for home-based breast expansion. However, these devices are expensive, providing an economic challenge for implementing AeroForm tissue expanders as an alternative to saline-based tissue expanders [15]. Whereas the features of AeroForm-based breast expanders are attractive, there is a need for ongoing efficacy and safety evaluation of their outcomes. The available literature is inconclusive to generate enough evidence for surgeons and patients. This is because of the insufficient number of randomized clinical trials, relatively small sample size of the published studies, and the short follow-up periods [16]. Furthermore, there is a demanding concern to provide a gradual, controlled, and comfort-guided tissue expansion process for breast reconstruction. Therefore, the current systematic review and meta-analysis study was carried out to reveal the usability, safety, and economic burden of AeroForm-based tissue expanders to reconstruct breasts.

## Materials and Methods

The current systematic review and meta-analysis study was executed in accordance with Preferred Reporting Items for Systematic Reviews and Meta-Analysis (PRISMA) guidelines [17] and the recommendations of Cochrane collaboration [18] (Supplementary Table.1). The study's methodology was documented in a protocol that was registered at the PROSPERO database (number; CRD42022300244).

### Data Source

An extensive systematic literature review was implemented from inception to 9 December 2021, using the following databases: PubMed, Google Scholar, Web of Science (ISI), Scopus, SIGLE, Virtual Health Library (VHL), NYAM, Controlled Trials (mRCT), Clinical trials, Cochrane Collaboration, WHO International Clinical Trials Registry Platform (ICTRP), and EMBASE. The following keywords were used in every possible combination; ‘Carbon Dioxide’, ‘Aeroform’, ‘Co2’, ‘Breast’, ‘Mammary’, ‘Reconstruction’,’ Expansion,’ Expanders’,’. No restrictions were employed on patients’ age, sex, ethnicity, language, race, or place.

The search strategy implemented controlled vocabulary terms under the criteria of each searched database. A further manual search was executed to distinguish all additional conceivable articles that are not indexed. The cross-referencing method was carried out until no other relevant articles were detected.

### Study Selection

All clinical studies that included female patients with breast cancer and subjected to AeroForm-based tissue expansion for breast reconstruction were included in the study. Furthermore, studies in which data were unattainable to be extracted, review articles, guidelines, non-human studies, case reports, letters, comments, editorials, posters, and book chapters were excluded. Two reviewers performed the title, abstract, and full-text screening process to disclose the potentially relevant articles that met the eligibility criteria. The discussion dissolved the contradiction between the reviewers. The screening process and the causes of article exclusion were documented using PRISMA Flow chart.

### Data Extraction

Two reviewers extracted the data in a well-structured Microsoft excel spreadsheet. The following study characteristics data were extracted from the finally included articles; the title of the included study, the second name of the first author, year of publication, study design, registration number, study period, and study region. Baseline patients' demographic characteristics were extracted, including patients' age, ethnicity, race, and body mass index (BMI). The data related to breast cancer were extracted consisting of breast cancer stage, previous mastectomy, previous radiotherapy, and history of neoadjuvant chemotherapy. The variables related to surgical procedure, which included the pattern of mastectomy, the timing of reconstruction, pocket location of the implant, implant size, intra-operative filling amount, and implant coverage, were extracted. The usability of AeroForm devices was assessed based on the treatment success rate, treatment failure rate, number of days to complete expansion, number of days to complete reconstruction, patients’ satisfaction, and surgeons’ satisfaction. The safety of the device was evaluated based on the breast-related and the device-related adverse events. The economic burden of AeroForm devices was assessed by extracting the overall cost with the expander, expected cost for surgery, quality-adjusted life years (QALYs) for successful surgery, QALYs gained, and incremental cost-effectiveness ratio (ICER). The data were extracted from graphs using WebPlotDigitizer software (https://automeris.io/WebPlotDigitizer/ ) [19].

### Risk of Bias and Quality Assessment

The risk of bias of the randomized clinical trials was evaluated based on the Cochrane Collaboration's tool for assessing the risk of bias. This tool is composed of seven items. These items assessed the random sequence generation, selection bias, performance bias, detection bias, attrition bias, reporting bias, and other possible causes of bias [20]. The quality of the observational studies was estimated using the National Institute of Health (NIH) quality assessment tool [21]. Based on this tool, the studies were categorized into good, fair, and bad when the score was <65%, 30-65%, > 30%, respectively. If the parameter was controlled, the domain was considered "Yes "and vice versa. The quality of the included economic evaluation studies was assessed using a custom checklist. This checklist is based on the items included in the CHEC Criteria List (The Drummond Checklist adapted from Drummond et al. Methods for the Economic Evaluation of Health Care Programmes) [22].

### Statistical Analysis

Weighted mean difference (WMD) or standardized mean difference (SMD) was used for analysing the continuous variables. Data reported in the form of median and range were converted to mean and standard deviation (SD) based on Hozo et al. equations [23]. The risk ratio (RR) or odds ratio (OR) with 95% confidence interval (CI) was used for analysing dichotomous variables. The satisfaction rate, procedure-related pain rate, and the rate of device removal were estimated by calculating the event rate and 95% CI for each study. This was succeeded by pooling the effect sizes of all studies to estimate the summary proportion with 95% CI. The fixed-effect model was implemented when a fixed population effect size was assumed; otherwise, the random-effects model was used. Statistical heterogeneity was evaluated using Higgins *I*^*2*^ statistic, at the value of > 50%, and the Cochrane Q (*Chi*^2^ test), at the value of *p* < 0.10 [24]. Publication bias was assumed in the presence of an asymmetrical funnel plot and based on Egger’s regression test (*P*-value <0.10) [25]. Data analysis was performed using Review Manager version 5.4 and Comprehensive Meta-Analysis v3 software [26, 27]. The significance was established at the value of *P* < 0.05.

## Results

**Fig. 1:**
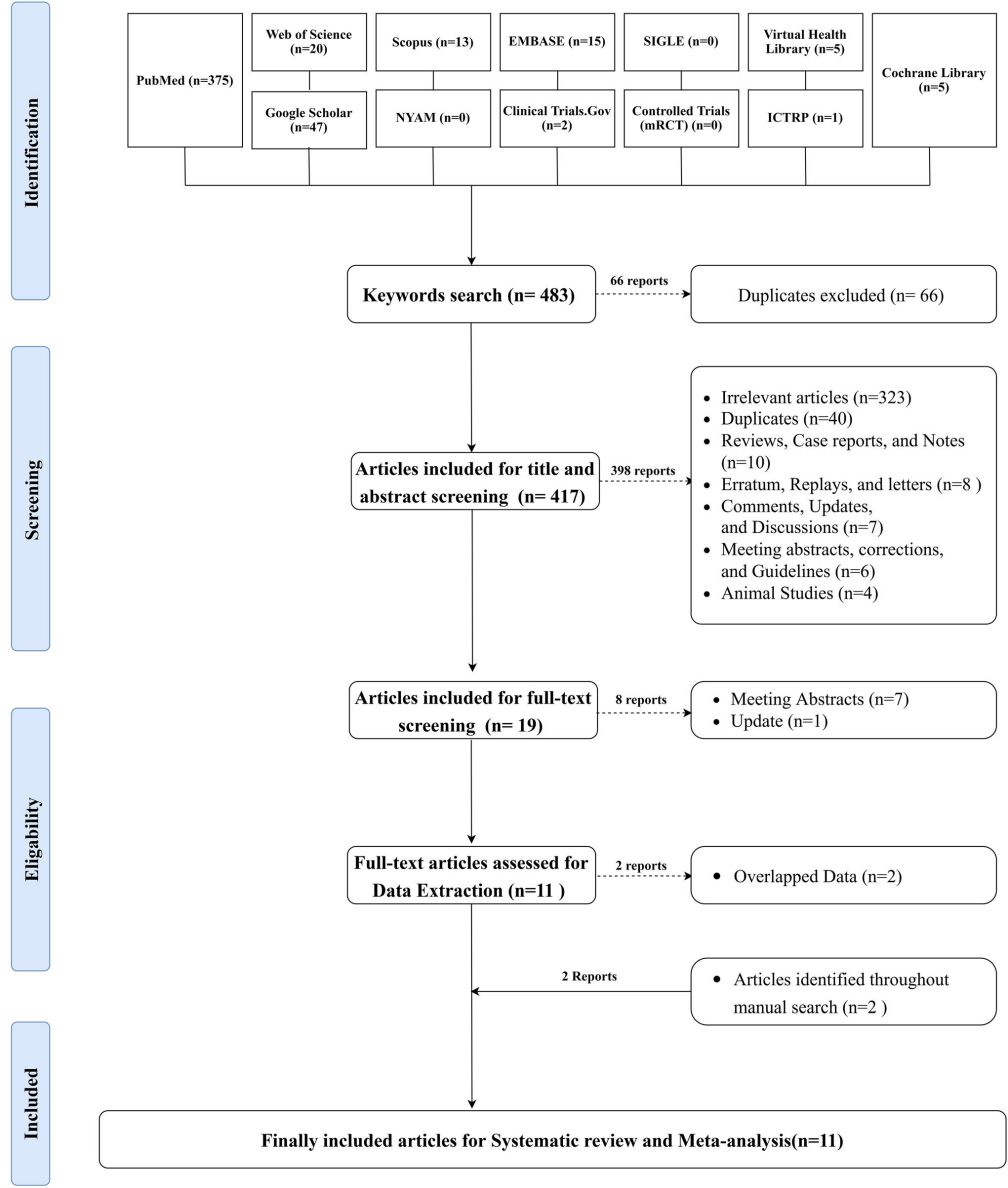
PRISMA Flow chart showing the process of the literature search, title, abstract, and full-text screening, systematic review, and meta-analysis.

The extensive literature search yielded 483 articles. Of them, 66 articles were excluded, being duplicates, leaving 417 studies eligible for the title and abstract screening. Out of them, 398 articles were excluded, yielding 19 eligible for full-text screening. Eleven articles were included for data extraction, from which two were excluded being overlapped data. Two studies were identified through the manual search, yielding eleven articles eligible for systematic review and meta-analysis. The searching strategy for each searched database is shown in Supplementary Table.2**.** The process of searching strategy, screening, and eligibility is shown in the PRISMA flow chart. (Fig. 1)

### Patients’ Demographic Characteristics

The present systematic review included eleven articles consisting of 748 patients. There were four articles of the observational design and three of the retrospective design. There were three randomized clinical trials. Seven articles included patients from the USA, while three included patients from Australia. The age of the included patients was 48.5 to 51 years in the AeroForm group and from 48.9 to 51.22 years among the saline group. The current systematic review included 1220 reconstructed breasts in which 530 (43.44%) breasts were reconstructed using AeroForm-based tissue expanders. Sixty-two patients had breast cancer stage II within the AeroForm group, relative to 25 patients within the saline group. Among the AeroForm group, 39 patients received previous radiotherapy, relative to 175 patients within the saline group. (Table 1)

**Table 1:** Demographic characteristics of the included studies

Study ID		Study Region	Study Design	Study Period	Sample Size		Number of breasts		Age (Years)		BMI (Kg/m^2^)	
					AeroForm	Saline	AeroForm	Saline	AeroForm	Saline	AeroForm	Saline
					Number	Number	Number	Number	Mean± SD	Mean± SD	Mean± SD	Mean± SD
1	Ascherman et al., 2016[29]	USA	RCT	October of 2011 and December of 2014	98	52	168	88	48.9 ± 9.83	51.1 ± 9.88	25.2 ± 4.08	24.8 ± 4.25
2	Ascherman et al., 2020[28]	USA	RCT	November 12, 2015, and September 20, 2016.	50	NR	86	NR	48.7*	NR	24.5 ± 3.57	NR
3	Connel et al., 2011[34]	Australia	prospective, open-label, feasibility clinical study	June 22, 2009, to September 30, 2009	7	NR	9	NR	NR	NR	NR	NR
4	Connel et al., 2014[35]	Australia	prospective, open-label, confirmatory clinical study	July of 2011 to June of 2012	33	NR	61	NR	NR	NR	24.6 ± 3.8	NR
5	Connel et al., 2015[30]	Australia	RCT	July 19, 2013 to March 26, 2014	21	NR	34	NR	49.7 ± 8.6	NR	26.1 ± 4.7	NR
6	Chopra et al., 2019a[32]	USA	Retrospective study	NR	47	68	74	111	49.1 ± 12.6	50.0 ± 10.5	27 ± 6	27 ± 6
7	Chopra et al., 2019b[31]	USA	Perspective decision Model	NR	458	NR	NR	NR	NR	NR	NR	NR
8	Hsieh et al., 2015[36]	Australia	Prospective Study	May to November 2013	10	NR	12	NR	NR	NR	NR	NR
9	Kelley et al., 2017[38]	USA	Prospective Study	NR	8	NR	NR	NR	NR	NR	NR	NR
10	Kraenzlin et al., 2020[33]	USA	Retrospective study	October 2016 to June 2018	37	132	57	210	48.5*	48.9*	NR	NR
11	Porter et al., 2019[37]	USA	Retrospective study	January 1, 2005, and February 5, 2019	185	21	281	51.0 ± 9.3	51.22 ± 11.51	23.0 ± 2.87	27.22 ± 6.07	NR

Study ID		Breast cancer stage								Previous Radiotherapy		Previous Neoadjuvant chemotherapy	
		0		I		II		III					
		AeroForm	Saline	AeroForm	Saline	AeroForm	Saline	AeroForm	Saline	AeroForm	Saline	AeroForm	Saline
		Number	Number	Number	Number	Number	Number	Number	Number	Number	Number	Number	Number
1	Ascherman et al., 2016[29]	22	11	31	17	33	14	5	2	3	21	6	NR
2	Ascherman et al., 2020[28]	9	NR	14	NR	9	NR	3	7	NR	14	NR	NR
3	Connel et al., 2011[34]	NR	NR	NR	NR	NR	NR	NR	NR	NR	NR	NR	NR
4	Connel et al., 2014[35]	NR	NR	NR	NR	NR	NR	NR	NR	NR	13	NR	NR
5	Connel et al., 2015[30]	2	NR	4	NR	6	NR	4	10	NR	16	NR	NR
6	Chopra et al., 2019a[32]	16	29	16	24	11	11	2	8	7	6	18	NR
7	Chopra et al., 2019b[31]	NR	NR	NR	NR	NR	NR	NR	NR	NR	NR	NR	
8	Hsieh et al., 2015[36]	NR	NR	NR	NR	NR	NR	NR	NR	NR	NR	NR	NR
9	Kelley et al., 2017[38]	NR	NR	NR	NR	NR	NR	NR	NR	NR	NR	NR	NR
10	Kraenzlin et al., 2020[33]	NR	NR	NR	NR	NR	NR	NR	2	26	16	26	NR
11	Porter et al., 2019[37]	NR	NR	NR	NR	NR	NR	NR	3	89	5	118	

*RCT* Randomized clinical trial, *BMI* Body mass index, * Data reported in the form of mean only, *NR* Non-Reported

The simple mastectomy was performed among 108 and 47 patients within AeroForm and saline groups. Furthermore, 160 patients were subjected to nipple-sparing mastectomy among the AeroForm group compared to 187 patients within the saline group. Immediate breast reconstruction was implemented among 233 patients among the AeroForm group, while delayed reconstruction was carried out among 48 patients. Of note, 458 implants were positioned at the subpectoral pocket, while 452 implants were positioned at the prepectoral pocket. The frequently used expander size was the small in 71 reconstructed breasts. The average follow-up period ranged from one month to 12 months. (Table 2)

**Table 2:** Breast surgeries related data and the results of the quality assessment of the included studies

Study ID		Mastectomy						Breast Reconstruction							
		Simple		Modified radical		Nipple Sparing		Unilateral		Bilateral		Immediate		Delayed	
		AeroForm	Saline	AeroForm	Saline	AeroForm	Saline	AeroForm	Saline	AeroForm	Saline	AeroForm	Saline	AeroForm	Saline
		Number	Number	Number	Number	Number	Number	Number	Number	Number	Number	Number	Number	Number	Number
1	Ascherman et al., 2016[29]	75	42	20	8	59	33	28	16	140	72	154	83	14	5
2	Ascherman et al., 2020[28]	28	NR	4	NR	47	NR	14	NR	72	79	NR	NR	7	NR
3	Connel et al., 2011[34]	NR	NR	NR	NR	NR	NR	NR	NR	NR	NR	NR	NR	NR	NR
4	Connel et al., 2014[35]	NR	NR	NR	NR	NR	NR	5	NR	28	NR	26	NR	3	NR
5	Connel et al., 2015[30]	NR	NR	NR	NR	4	NR	8	NR	13	NR	12	NR	6	NR
6	Chopra et al., 2019a[32]	5	5	1	1	24	30	NR	NR	NR	NR	36	65	11	3
7	Chopra et al., 2019b[31]	NR	NR	NR	NR	NR	NR	NR	NR	NR	NR	NR	NR	NR	NR
8	Hsieh et al., 2015[36]	NR	NR	NR	NR	NR	NR	NR	NR	NR	NR	5	NR	7	NR
9	Kelley et al., 2017[38]	NR	NR	NR	NR	NR	NR	NR	NR	NR	NR	NR	NR	NR	NR
10	Kraenzlin et al., 2020[33]	NR	NR	NR	NR	26	124	NR	NR	NR	NR	NR	NR	NR	NR
11	Porter et al., 2019[37]	NR	NR	0	18	NR	NR	NR	NR	NR	NR	NR	NR	NR	NR

Study ID		Expander Size			Implant coverage				Quality Assessment	
					Acellular dermal matrix		Latissimus dorsi			
		Small	Medium	Large	AeroForm	Saline	AeroForm	Saline	%	Decision
		Number	Number	Number	Number	Number	Number	Number		
1	Ascherman et al., 2016[29]	NR	NR	NR	108	52	2	2	–	–
2	Ascherman et al., 2020[28]	40	40	6	73	NR	1	NR	–	–
3	Connel et al., 2011[34]	3	4	2	NR	NR	7		–	–
4	Connel et al., 2014[35]	NR	NR	NR	NR	NR	61	61	66.6%	Good
5	Connel et al., 2015[30]	21	13	0	0	NR	30		66.6%	Good
6	Chopra et al., 2019a[32]	NR	NR	NR	NR	NR	3	2	66.6%	Good
7	Chopra et al., 2019b[31]	NR	NR	NR	NR	NR	NR	NR	–	–
8	Hsieh et al., 2015[36]	7	5	0	NR	NR	NR	NR	58.83%	Fair
9	Kelley et al., 2017[38]	NR	NR	NR	NR	NR	NR	NR	50%	Fair
10	Kraenzlin et al., 2020[33]	NR	NR	NR	57	210	NR	NR	75%	Good
11	Porter et al., 2019[37]	NR	NR	NR	NR	NR	NR	NR	66.6%	Good

*NR* Non-Reported

### Risk of Bias and Quality Assessment

**Fig. 2:**
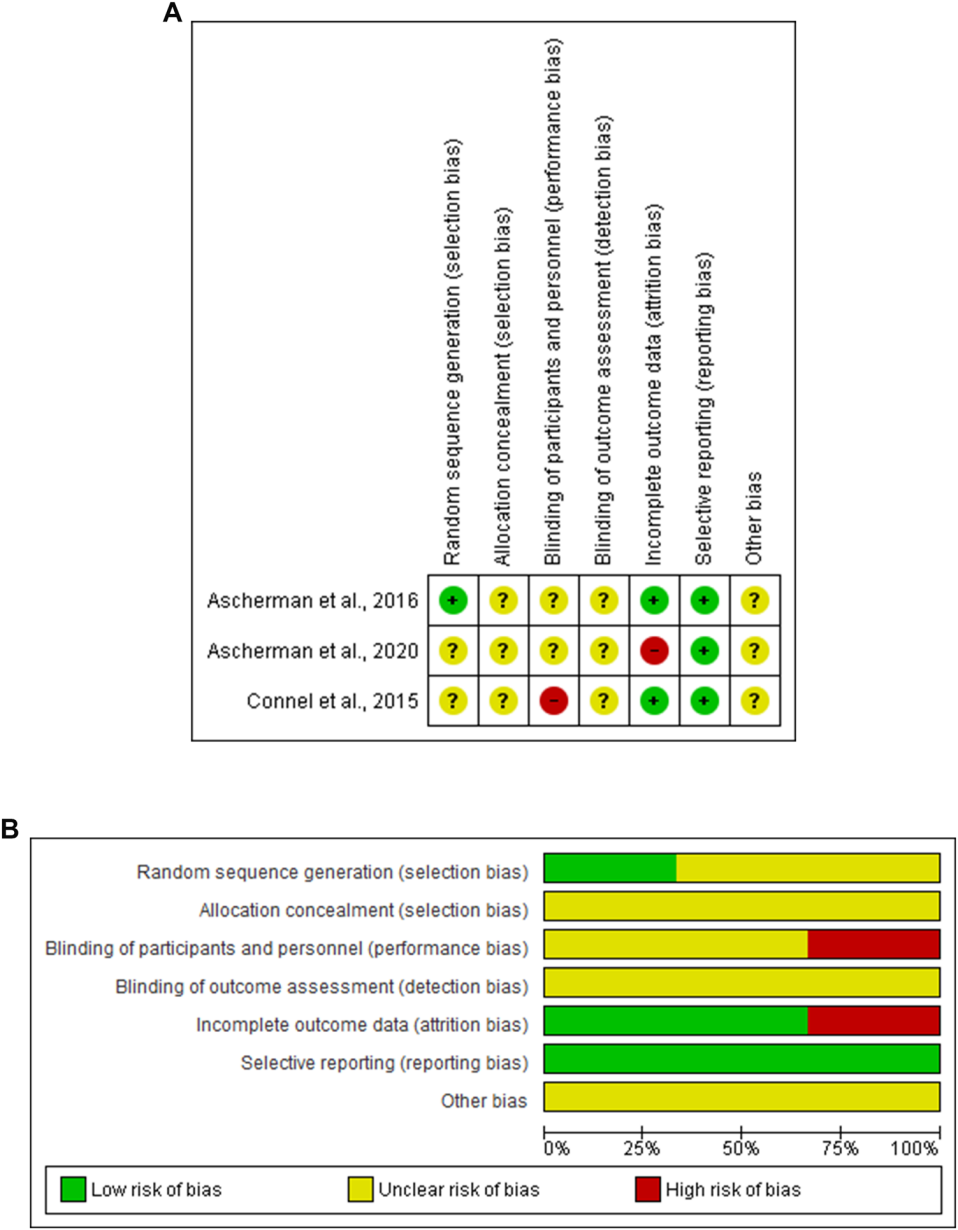
**A** Risk of bias graph, **B** Risk of bias summary: review authors' judgements about each risk of bias item presented as percentages across all included studies.

Three studies [28–30] were assessed for risk of bias based on the Cochrane Collaboration's tool. One study [29] reported a low risk of selection bias, whereby the three studies reported an unclear risk of detection bias. The three studies reported a low risk of reporting bias, while one study [28] showed a high risk of attribution bias. Based on the NIH quality assessment tool, there were five articles of good quality and three articles of fair quality. Based on The Drummond Checklist adapted, Chopra et al. [31] fulfilled the criteria for study design and partially fulfilled the criteria related to data collection and analysis and interpretation of results. (Fig. 2 and Table 2)

### Efficacy and Usability of the AeroForm Tissue Expanders

**Fig. 3:**
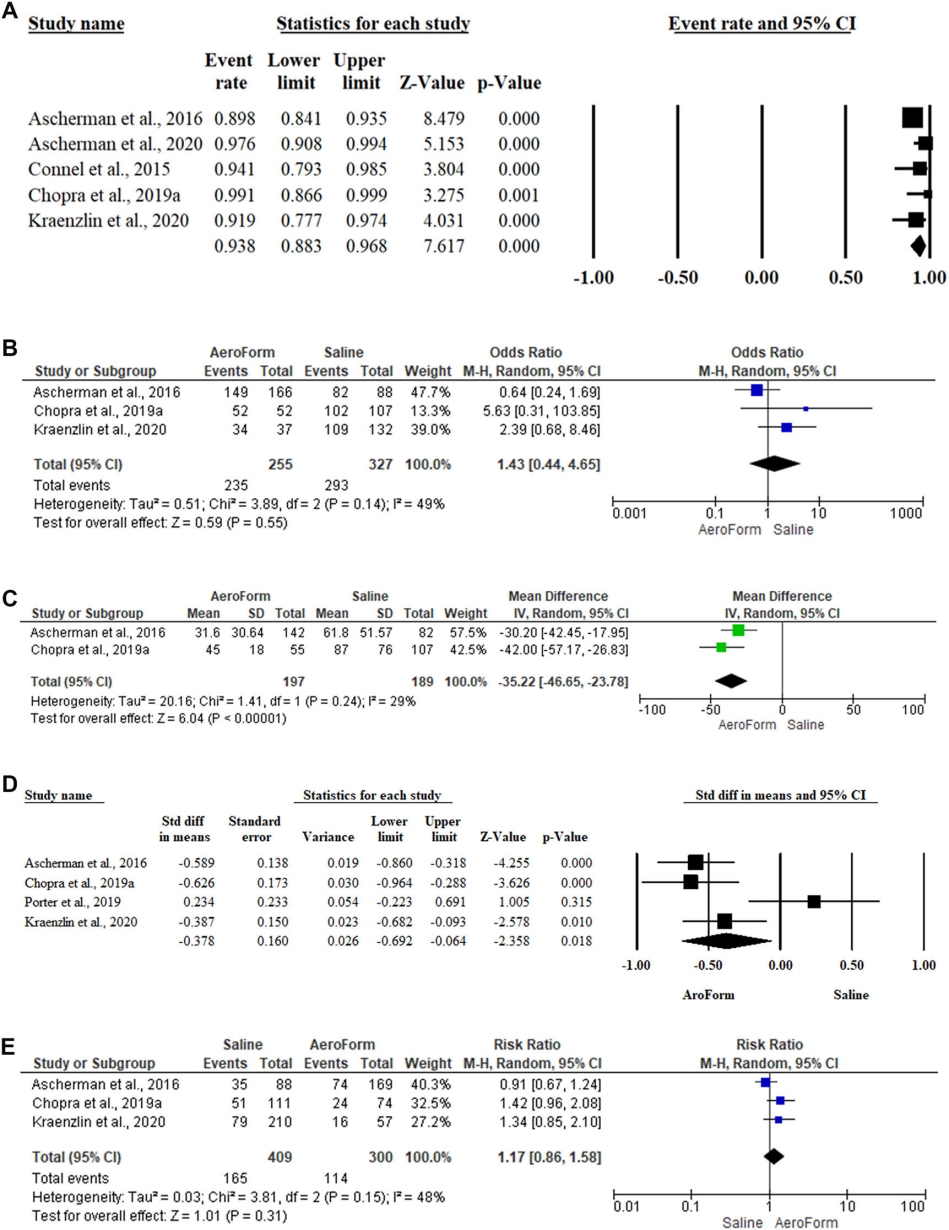
Forest plot of summary analysis of the **A** Treatment success rate and 95% CI among patients subjected to AeroForm-based tissue expansion **B** Odds ratio and 95% CI of the treatment success between AeroForm-based and Saline-Based Tissue expanders groups. **C** Standardized Mean difference and 95% CI of the mean duration to complete expansion between AeroForm-based and Saline-Based Tissue expanders groups **D** Mean difference and 95% CI of the mean duration to complete reconstruction between AeroForm-based and Saline-Based Tissue expanders groups **E** Risk ratio and 95% CI of the breast-related adverse events between AeroForm-based and Saline-Based Tissue expanders groups. Size of the green, blue, and black squares is proportional to the statistical weight of each trial. The grey diamond represents the pooled point estimate. The positioning of both diamonds and squares (along with 95% CIs) beyond the vertical line (unit value) suggests a significant outcome (IV = inverse variance).

The success rate of Aeroform-based tissue expansion was reported within five articles [28–30, 32, 33]. Among 718 reconstructed breasts, 347 breasts were reconstructed optimally with a success rate of 93.8% (95CI% 88.3%, 96.8%, p<0.001). Compared to saline-based tissue expansion, patients who received AeroForm-based breast expansion were 1.43 times more liable to achieve successful implant-based breast reconstruction (OR 1.43; 95% 0.44, 4.65; P=0.55). (Fig. 3A and 3B)

#### Number of Days to Complete Expansion

Seven articles included 393 reconstructed breasts reported the average number of days to complete expansion among the Aeroform group [28–30, 32, 34–36]. The mean number of days was 14.25 to 45 days and 61.8 to 87 days among the Aeroform and saline groups. In the random-effects model (*I*^2^=29%, *P*=0.24), patients subjected to AeroForm-based breast expansion had statistically significant shorter duration to complete breast expansion (MD −35.22; 95% −46.65, −23.78; *P*<0.001). (Fig. 3C)

#### Number of Days to Complete Reconstruction

The number of days to complete breast reconstruction in the AeroForm group was evaluated among 448 reconstructed breasts within seven studies [28–30, 32, 33, 35, 37]. The mean number of days was 90 to 135.4 days within the AeroForm group compared to 90.9 to 181.7 days within the saline group. In the random-effects model (*I*^2^=68%, *P*=0.024), the mean number of days to complete reconstruction was significantly shorter among patients subjected to AeroForm-based breast expansion (MD −30.511; 95% −54.659, −6.636; *P*=0.013). (Fig. 3D)

#### Surgeons’ Satisfaction and Usability

Three studies [28–30], which included 273 reconstructed breasts, reported the surgeons’ satisfaction rate regarding the AeroForm devices. The overall satisfaction rate of the aesthetic results was 64.6% (95%CI; 53.8% to 74%, *P*=0.008), in which 177 breasts were reconstructed optimally. Regarding the surgeon satisfaction rate of the device usage, pooled data from three studies [28, 29, 34] revealed a rate of 87.9% (95%CI; 59.8% to 95.8%, *P*=0.001). (Supplementary Fig. 1A and 1B)

#### Patients’ Satisfaction and Usability

Five articles, including 310 reconstructed breasts, reported the patients’ satisfaction rate regarding the results of AeroForm-based breast expansion [28–30, 34, 35]. The overall satisfaction rate of the aesthetic results was 81.4% (95%CI; 60.3% to 92.6%, P=0.006). The usability of the device was assessed within three articles [30, 35, 38], including 59 reconstructed breasts with a satisfaction rate of 96.66% (95%CI; 87.4% to 99.1%, *P*<0.001). Three studies [29, 30, 35] included 260 patients reported the incidence of home-expansion-related procedure pain. In the random-effects model (*I*^2^=25%, *P*=0.26), the rate of procedure-related pain was 7.4% (95% CI: 4.2% to 12.7%. *P*<0.001) among the AeroForm group. (Supplementary Fig. 1C, 1D and 1E)

### Safety of the AeroForm Tissue Expanders

#### Breast-Related Adverse events

**Fig. 4:**
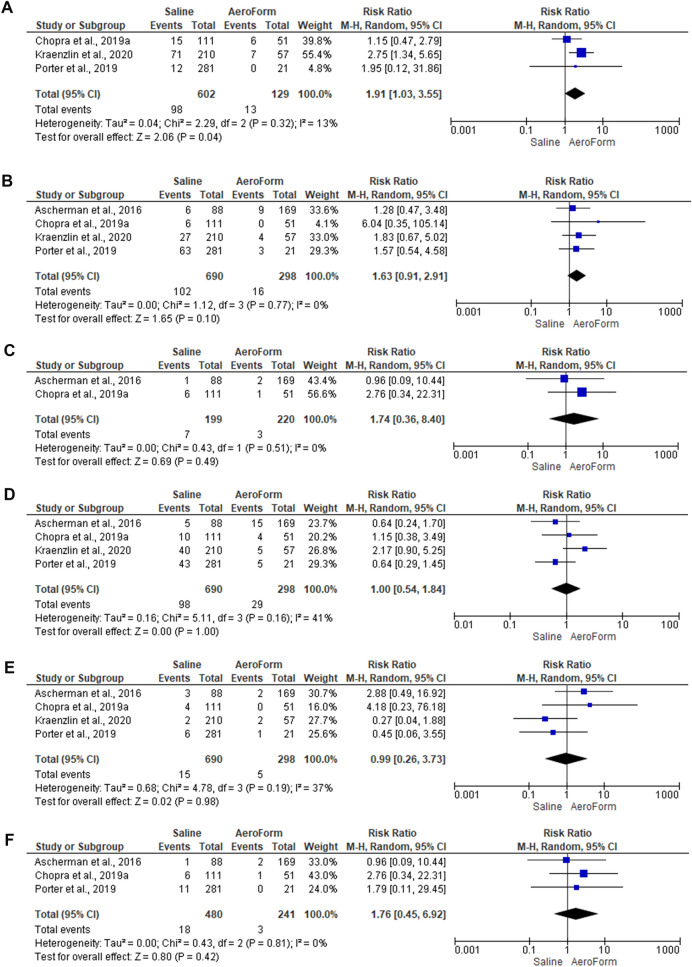
Forest plot of summary analysis of the risk ratio and 95% CI of **A** The risk of the mastectomy flap necrosis risk between AeroForm-based and Saline-Based Tissue expanders groups. **B** The risk of the post-operative wound infection risk between AeroForm-based and Saline-Based Tissue expanders groups **C** The risk of the wound dehiscence between AeroForm-based and Saline-Based Tissue expanders groups **D** The risk of the seroma between AeroForm-based and Saline-Based Tissue expanders groups **E** The risk of the hematoma between AeroForm-based and Saline-Based Tissue expanders groups. **F** The risk of the device extrusion between AeroForm-based and Saline-Based Tissue expanders groups. Size of the blue squares is proportional to the statistical weight of each trial. The grey diamond represents the pooled point estimate. The positioning of both diamonds and squares (along with 95% CIs) beyond the vertical line (unit value) suggests a significant outcome (IV = inverse variance).

Three studies [29, 33, 39], included 709 reconstructed breasts, compared the breast-related adverse events between AeroForm and saline groups. In the random-effects model (*I*^2^=48%, *P*=0.15), patients who received saline-based breast reconstruction were 1.17 times at high risk of developing breast-related adverse events. No statistically significant difference was observed between both groups (RR 1.17; 95% 0.86, 1.58; *P*=0.31). Furthermore, patients who received saline-based tissue expansion were 1.91 times more susceptible to developing mastectomy flap necrosis (RR 1.91; 95% 1.03, 3.55; *P*=0.04) than patients subjected to AeroForm-based breast expansion. Patients received saline-based tissue expansion were 1.63 times more vulnerable to develop post-operative wound infection (RR 1.63; 95% 0.91, 2.91; *P*=0.1). (Fig. 3E, 4A, and 4B)

Two studies [29, 32], including 419 reconstructed breasts, evaluated the risk of wound dehiscence between AeroForm and saline-based tissue expanders. In the random-effects model (*I*^2^=0%, *P*=0.51), patients subjected to saline-based tissue expansion were 1.74 times more susceptible to develop wound dehiscence (RR 1.74; 95% 0.36, 8.40; *P*=0.49). There was an equal risk of seroma (RR 1.00; 95% 0.54, 1.84; *P*=1) and hematoma (RR 0.99; 95% 0.26, 3.73; *P*=0.98) between AeroForm and saline-based breast expansion groups. (Fig. 4C, 4D, and 4E)

#### Device -Related Adverse Events

Three studies [29, 32, 37] included 721 reconstructed breasts assessed the extrusion rate between Aeroform and saline-based tissue expanders. In the random-effects model (*I*^2^=0%, *P*=0.81), saline-based tissue expanders were 1.76 times more susceptible to extrusion (RR 1.76; 95% 0.45, 6.92; *P*=0.42), relative to AeroForm-based expanders. Two studies [30, 37] reported the rate of expander removal among 55 reconstructed breasts. Out of them, the expander was removed within three reconstructed breasts with a rate of 5.5% (95%CI 1.8% to 15.7%). (Fig. 4F and supplementary Fig. 1F)

### Economic Evaluation of the AeroForm Tissue Expanders

Two studies [31, 37] included 760 reconstructed breasts reported the economic burden of the AeroForm-based breast reconstruction. The overall costs of the AeroForm-based tissue expanders ranged from 2360 to 2552.02 US dollars, while the costs ranged from 1882 to 2090.59 US dollars for saline-based tissue expanders. The expected costs for breast reconstruction surgery were 3210.16 and 3458.71 US dollars for breasts reconstructed by AeroForm and saline tissue expanders. The QALYs Gained from AeroForm-based tissue expanders were 0.00122 with an ICER of -206,901.36.

## Discussion

The AeroForm-based tissue expanders have been evolved as recent breast reconstruction innovation. However, the available literature is inconclusive regarding the effectiveness and usability of AeroForm tissue expanders, making it challenging to draw firm conclusions. Accordingly, this meta-analysis was conducted to draw conclusive evidence from the available literature regarding the outcomes of AeroForm devices in breast reconstruction. The present systematic review revealed that Aeroform-based tissue expanders are associated with promising usability and safety outcomes. These devices are associated with a higher success rate and shorter duration to achieve complete expansion and reconstruction relative to saline-based expanders. Furthermore, most surgeons and patients were satisfied with the breasts' final appearance and the ability to control the process of tissue expansion. Patients subjected to breast reconstruction using Aeroform-based tissue expanders were at low risk of developing breast-related and device-related adverse events. This includes a low rate of mastectomy flap necrosis, wound infection, wound dehiscence, and device extrusion. Aeroform-based tissue expanders are cost-saving to reconstruct the breasts, with clinical benefits exceeding the obtained from saline-based tissue expanders. These findings indicate that AeroForm devices are a valuable choice for implant-based breast reconstruction.

AeroForm-based tissue expanders are associated with quicker implant exchange and final reconstruction. This is because of the ability of the patients to expand these devices gradually at home with 210 ccs of gas per week. This volume was larger than the weekly bolus dose of conventional saline-based tissue expanders. The use of a remote controller to adjust the expansion process made breast reconstruction more convenient and more accessible. This offered the surgeon to exchange the implant before requiring post-mastectomy radiotherapy. In this respect, Moni et al. reported that radiation dose could be delivered with acceptable limits even with the metal reservoir and the gas chamber of the AeroForm-based tissue expanders [40]. However, Lim et al. reported that radiotherapy should be delivered after placing the permanent implants as AeroForm devices create dose uncertainties, disrupting the chest wall-metal interface [41]. Noteworthy, radiotherapy is integral for most women who underwent mastectomy and breast reconstruction. Therefore, the optimal timing of radiation therapy needs to be detected, and the effects of radiation on the AeroForm-based devices require further evaluation.

Mastectomy flap necrosis and wound infection have devastating consequences in breast reconstruction. These complications necessitate tissue expander removal, delay reconstruction, and postpone adjuvant therapies. Subsequently, they require further operative procedures associated with increased costs and poor aesthetic outcomes [42, 43]. In the present study, the risk of mastectomy flap necrosis was approximately half among patients who underwent AeroForm-based tissue expansion. This is because of the gradual filling of these devices, low expander weight, and equal distribution of this weight within the implant. These factors reduced the risk of sudden ischaemic insults to the mastectomy skin flaps, which is more prominent in saline-based tissue expanders. This is because of the weekly bolus filling of the saline expanders causes noticeable abrupt volume changes. The sudden changes in the expander volume enhance the pressure on the flap with a devastating cascade of events resulting from a progressive increase in weight, leading to flap necrosis [44]. This also explained the high risk of wound dehiscence and extrusion associated with saline-based tissue expanders relative to AeroForm devices. Furthermore, leaving the AeroForm tissue expanders relatively unfilled in the perioperative period allowed enough time for the mastectomy skin flaps to recover from the stress of surgery without further vascular stress [45]. In the current systematic review, the risk of post-operative wound infection was relatively low among patients who underwent AeroForm-based tissue expanders. This is because AeroForm devices require no percutaneous needlesticks. This reduced the risk of iatrogenic infection and device rupture. Moreover, the home-based expansion of AeroForm devices reduced the burden of frequent clinic visits associated with saline expanders, decreasing the risk of hospital-acquired infection. This minimized the utilization of healthcare resources, which expanded surgeons' ability to care for more patients [16].

The present study justified the economical use of AeroForm tissue expanders for breast reconstruction. AeroForm-based tissue expanders technology offered the advantages of convenience and cost-saving for breast reconstruction. These devices reduced the economic burden of saline-based tissue expansion counterpart the frequent clinic visiting for expansion. Additionally, AeroForm-based tissue expanders reduced the healthcare costs of post-operative complications associated with saline-based tissue expanders [16, 46]. However, a major disadvantage of the AeroForm devices is the inability to remove the gas from the expander. This may lead to device overfilling and ischaemic necrosis. Another drawback is that the expansion process cannot be reversed except by puncturing the expander, which necessitates device removal [16]. Consequently, the oncologists need to adjust the radiotherapy dose to mitigate the presence of gas within the Aeroform devices. These devices could temporarily expand with increasing altitude during air travel. Accordingly, the dose of the gas should be reduced a few weeks before the trip to allow more space for expansion during travelling [16]. Aeroform-based tissue expanders are textured implants. Therefore, further studies are mandatory to evaluate the risk of anaplastic large cell lymphoma associated with AeroForm tissue expanders.

This systematic review and meta-analysis gathered the rapidly emerging controversial evidence regarding the impact of AeroForm-based tissue expanders on breast reconstruction. However, some limitations should be considered while interpreting the yielded evidence in the present study. Many of the included articles were observational designs, revealing a potential risk of selection bias. All the included studies included patients from the USA or Australia, making the generalization of the results is questionable. Consequently, the long-term impact of the AeroForm devices on the outcomes of breast reconstruction has not been evaluated comprehensively in the included studies.

## Conclusions

AeroForm-based tissue expanders represent a new era of breast reconstruction. These devices provided an earlier transition to exchange for the permanent implant with a convenient and comfortable expansion process. This was associated with a high satisfaction rate for patients and surgeons regarding the device's aesthetic outcomes and usability. Additionally, AeroForm-based tissue expanders were associated with a lower risk of adverse events, justifying the use of these devices as a cost-saving alternative to saline-based tissue expanders.

**Supplementary Fig. 1** Forest plot of summary analysis of the event rate and 95% CIs of **(A)** The surgeons’ satisfaction rate of the aesthetic results, **(B)** The surgeons’ satisfaction rate of the usability of AeroForm-based tissue expanders, **(C)** The patients’ satisfaction rate of the aesthetic results, **(D)** The patients’ satisfaction rate of the usability of AeroForm-based tissue expanders, **(E)** The rate of procedure-related pain, **(F)** The rate of expander removal. Size of the black squares is proportional to the statistical weight of each trial. The grey diamond represents the pooled point estimate. The positioning of both diamonds and squares (along with 95% CIs) beyond the vertical line (unit value) suggests a significant outcome (IV = inverse variance).

## Supplementary Information

Below is the link to the electronic supplementary material.

Supplementary file1 (TIF 29059 KB)

Supplementary file2 (DOCX 31 KB)

## Declarations

### Human and Animal Participants

This article does not contain any studies with human participants or animals performed by any of the authors.
