# Supplementary figures and images for: The Impact of Aeroform Tissue Expanders on the Outcomes of Implant-Based Breast Reconstruction; A Systematic Review and Meta-Analysis
Source: Aesthetic Plast Surg. 2022 May 13;47(1):130–43. doi: 10.1007/s00266-022-02901-y (PMC9944028; doi:10.1007/s00266-022-02901-y)

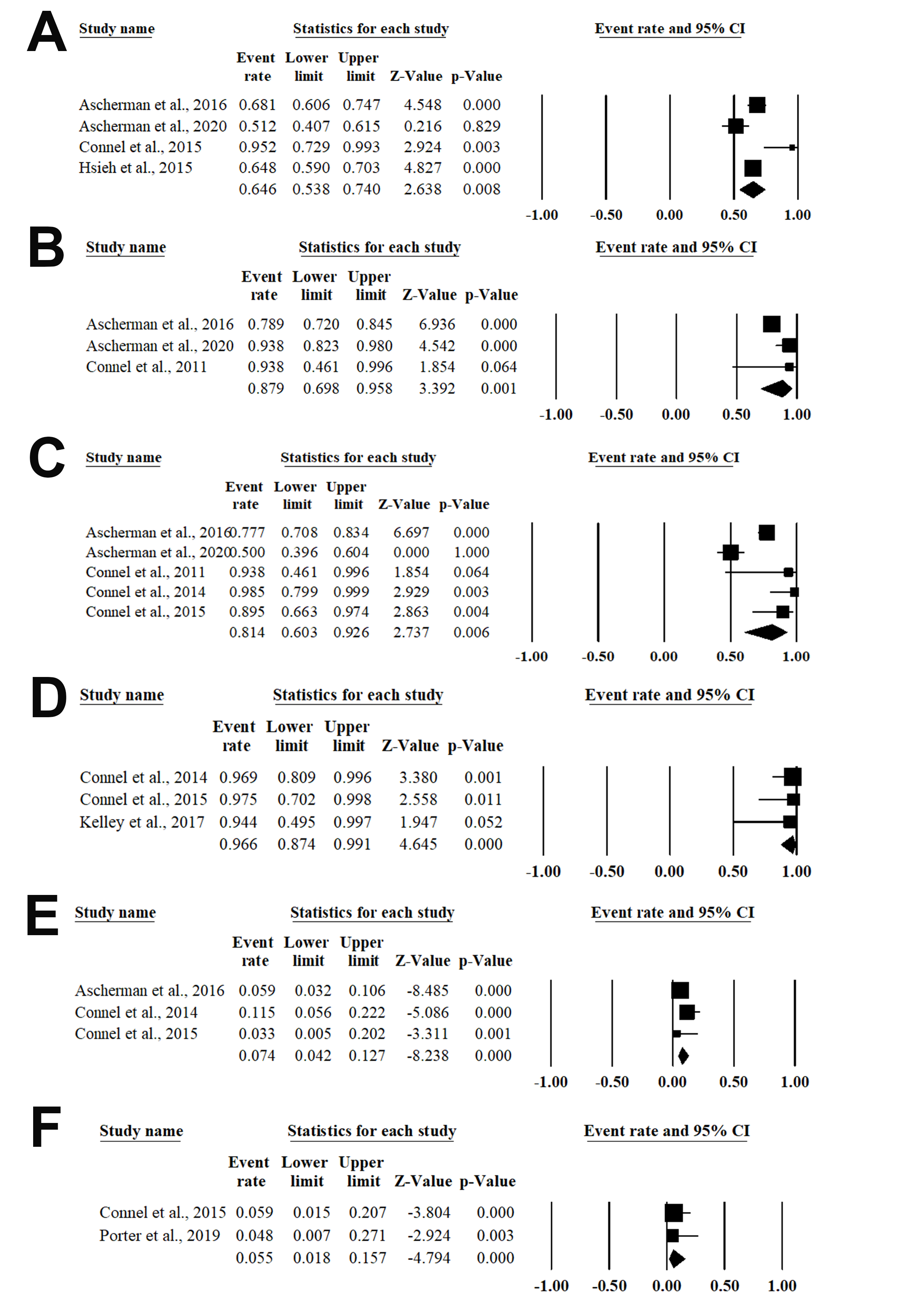

Supplement: Supplementary file 1 — Supplementary file1 (TIF 29059 KB) [file 266_2022_2901_MOESM1_ESM.tif]
